# Liquid Level Sensor Based on a V-Groove Structure Plastic Optical Fiber

**DOI:** 10.3390/s18093111

**Published:** 2018-09-14

**Authors:** Chuanxin Teng, Houquan Liu, Hongchang Deng, Shijie Deng, Hongyan Yang, Ronghui Xu, Ming Chen, Libo Yuan, Jie Zheng

**Affiliations:** 1 Photonics Research Centre, Guilin University of Electronic Technology, Guilin 541004, China; xinchuanteng@126.com (C.T.); houquanliu@163.com (H.L.); denghongchang86@163.com (H.D.); sdeng_guet@163.com (S.D.); yhy.gl@126.com (H.Y.); csxrh@163.com (R.X.); m_chen@126.com (M.C.); 2 Guangxi Key Laboratory of Optoelectronic Information Processing, Guilin University of Electronic Technology, Guilin 541004, China; 3 State Key Laboratory on Integrated Optoelectronics, College of Electronic Science and Engineering, Jilin University, Changchun 130012, China

**Keywords:** plastic optical fiber, liquid level sensor, V-groove structure

## Abstract

A high sensitivity and easily fabricated liquid level sensor based on the V-groove structure plastic optical fiber (POF) was described. In the design, the V-groove structure on the POF is produced by using a die-press-print method, which effectively reduces the complexity of the fabrication process and makes it easier for mass production of liquid level sensors. This greatly enhances the usefulness of the proposed sensor in cost effective liquid level sensing applications. The transmission characteristic of the POF could be changed when the V-groove structure was immerged or emerged by the rising or falling liquid. The liquid level sensing performances for the sensor probes with different structural parameters were investigated, and the sensor performances for the liquids with different refractive indices and the sensor dynamic response were also tested. Experimental results show that the sensor’s sensitivity can reach 0.0698 mm^−1^, with a resolution of 2.5 mm. Results also show that the sensor has a fast response time of 920 ms.

## 1. Introduction

Liquid level measurements play an important role in various applications, such as fuel-level detection in tanks, liquid level monitoring for the storage and transportation of chemical products, and leakage monitoring in medical instruments and industrial process, etc. To date, many kinds of liquid level sensing techniques have been reported, including electrical and optical technologies. Compared with traditional electrical counterparts, the optical liquid level sensor is a safer option as they do not generate electric sparks at work and can resist the corrosion of chemical reagents. Thanks to the development of optical fiber technology in recent decades, optical fibers can be designed as liquid level sensors [1,2,3,4]. Optical fibers based liquid level sensors are compact, lightweight, immune to electromagnetic interference, suitable for working in harsh environments, and are available for remote sensing and distributed measurement, which makes them good candidates for the measurement of flammable fluids or corrosive chemicals. Different designs of optical fibers based liquid level sensors have been proposed, including the fiber Mach-Zehnder interferometer [5], fiber surface plasmon resonance [6], fiber Fabry–Perot cavity [7], fiber long-period gratings [8], and so on. Those sensors, however, require complicated processes to fabricate and their sensing systems are very expensive.

Recent development of plastic optical fibers (POFs) makes them available for sensing fields [9,10]. Compared with glass counterparts, POFs are low cost, more flexible, easier to handle, and enable operation at a visible wavelength, which makes them an ideal fiber for developing low-cost liquid level sensors. Several POFs based liquid level sensors have been reported in the literature [11,12,13,14,15,16]. Lomer et al. proposed a bent side-polished POF for liquid level sensing [12], however, the sensing head was embedded in a Plexiglas block, which increases the device size. Zhang et al. employed twisted ordinary commercial POFs for liquid level sensing [13], however, the coupling efficiency of the ordinary bare fibers is too low, which leads to a low signal to noise ratio. Lin et al. presented a multi-point liquid level sensor using a group of POF segments [14], however, the POF segments were difficult to align. Paulo et al. proposed a POF with a grooves structure for liquid level sensing, howver, it is difficult to fabricate the grooves with the same depth, and to get a high sensing resolution [15].

In this paper, we present a high sensitivity and simple liquid level sensor by using a POF with a V-groove structure that was fabricated on the surface of a POF by a metal mould using a simple die-press-print method. When the V-groove structure on the POF is immerged or emerged by the rising or falling liquid, the coupling characteristics of the transmitted light will be altered, which will lead to a changed output optical power. The changes of the liquid level can be measured by monitoring the POF output optical power variations. The proposed sensor is compact, has a simple structure, is easy to fabricate, and its resolution can be set easily by changing the V-groove structure parameter. These make it very attractive for liquid level sensing applications requiring compact and low cost solutions.

## 2. Sensor Fabrication and Operation Principles

**Figure 1:**
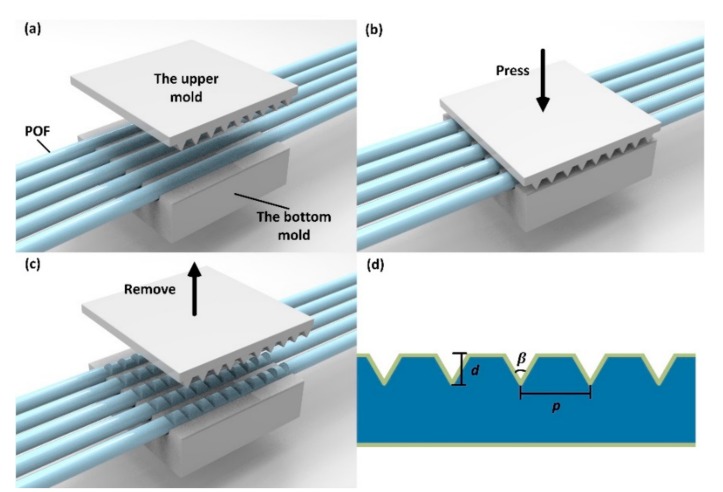
The schematics of the preparation of the sensor probes (**a**), (**b**), (**c**), and the V-groove structure on the plastic optical fiber (POF) (**d**), where *d* is the V-groove depth, *β* is the V-groove angle, and *p* is the V-groove pitch.

A commercial step-index POF (DD-1000, Jiangxi Daishing POF Co., Ltd., Jinggangshan, China) was used to fabricate the sensing probe. The core material of the POF is polymethyl methacrylate (PMMA) with a diameter of 980 μm, refractive index of 1.49, and thermo-optic coefficient of −1.15 × 10^−4^ °C^−1^, while the cladding material is a fluorinated polymer with a thickness of 10 μm, a lower refractive index of 1.41, and thermo-optic coefficient of −3.50 × 10^−4^ °C^−1^. The POF sensor probe was fabricated by a simple mechanical die-press-print method, as shown in Figure 1a–c. The POFs are placed into the U-shape slots (the diameter of the slot is about 1 mm) of the bottom metal mould, and an upper metal mould with a V-groove structure is pressed on the POFs, with the V-groove structure of the mould being perpendicular to the fiber axis. By keeping this state for a few seconds before removing the mould, the V-groove structure could be transferred onto the POFs. The structure parameters could be altered, with the V-groove pitch and angle changed using metal moulds with a different V-groove pitch and angle, respectively. The V-groove depth on the POF could be tuned by controlling the pressure and it was measured by using an optical microscope. The schematic of the V-groove structure on the POF is shown in Figure 1d. This preparation method is suitable for mass production.

The V-groove structure on the POF will cause the higher order propagating modes to refract into the cladding and radiate out of the fiber at one side of the V-groove structure, while some of them could couple back to the POF at the other side of the structure. The power of coupled light is dependent on the medium’s refractive index and the structural parameters of the V-groove. When all the V-groove structure is initially exposed in air whose refractive index is low, the light coupling between every V-groove structure is weak, thus the output power at the POF end is low. As the liquid level increases, the V-groove structure will be filled with the liquid, and the refractive index of the medium in the V-groove region increases. As a result, the light coupling is not so weak any more, and the output light power is enhanced accordingly. This can be explained as follows.

**Figure 2:**
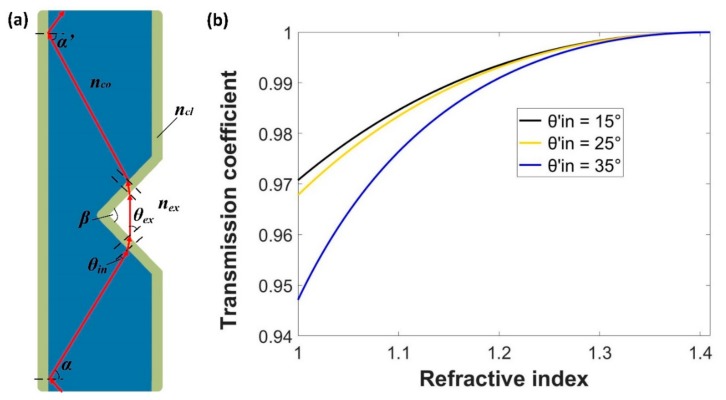
Diagram of the POF with one V-groove structure (**a**), and the effect of the refractive index of the medium in the V-groove region on the transmission coefficient for three different incident angles of *θ′_in_* (**b**).

For one V-groove structure (sensing area) situation, as shown in Figure 2a, *α* is the incident angle of a light ray at the core-cladding interface of the POF, *β* is the V-groove angle, and *θ_in_* is the incident angle at the V-groove region. It is found that the incident angle at the V-groove region is decreased, which will lead to more light power to go into the cladding or radiate out of the fiber. For a light ray that transmits from the POF cladding into the V-groove region medium (the polarization is ignored), the transmission coefficient, *T*, can be expressed as [14]:

(1) $$\;T=0.5\frac{\sin\left(2\theta_{in}^{\prime }\right)\sin\left(2\theta_{ex}\right)}{\sin^{2}\left(\theta_{in}^{\prime }+\theta_{ex}\right)}\left[1+\frac{1}{\cos^{2}\left(\theta_{in}^{\prime }-\theta_{ex}\right)}\right]\;$$

where *θ′_in_* is the incident angle at the cladding and the external medium interface, *θ_ex_* = arcsin[*n_cl_*sin(*θ′_in_*)/*n_ex_*], is the corresponding refraction angle at the cladding-medium interface in the V-groove region, and *n_ex_* is the refractive index of the external medium in the V-groove region. It can be found that the transmission coefficient, *T*, is expressed as a function of the incident angle, *θ′_in_*, and the refractive index of the external medium, *n_ex_*.

Figure 2b shows the relation between *T* and *n_ex_* ranging from 1 to 1.41 for different incident angles. It is found that the value of *T* increases when the incident angle decreases at the cladding-medium interface, and it is also found that as the refractive index of the external medium in the V-groove region increases, *T* increases. If the refractive index of the surrounding medium is low (e.g., n_air_ = 1), some of the light power could still be guided in the fiber cladding, in this case, the value of *T* decreases. While for the surrounding medium with a higher refractive index, for example 1.41, which is the same as the refractive index of the fiber cladding, all the modes guided through the cladding are refracted outside the fiber, so *T* increases. The situation is the same for the light coupled from the V-groove region into the POF. The liquid in the V-groove region could decrease the reflection at the external medium-cladding interface and make more light power couple back to the POF. Thus, when the V-groove structure is immersed by the rising liquid, whose refractive index is higher than that of air, more light power could be transported from one side of the V-groove structure to the other side, which makes the output power increase accordingly. Thus, it is possible to measure liquid-level changes by employing the probe with a cascading V-groove structure. The total output power of the POF probe is the sum of all the light rays propagated in the initial POF, which refract into the fiber cladding, undergo coupling at all the V-groove regions, and finally reach the last POF output end. Of course, some of the light rays will be directly lost in the V-groove regions.

## 3. Experimental Results and Discussion

**Figure 3:**
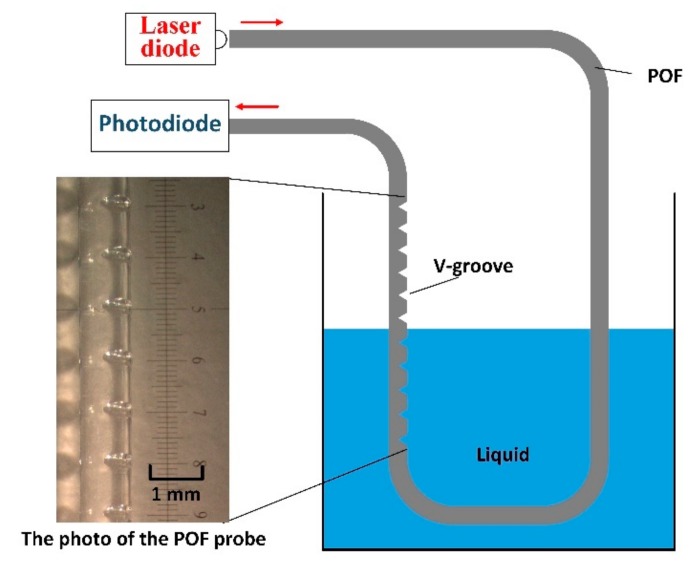
Schematic diagram of the experimental setup.

Figure 3 shows the experimental setup of the proposed liquid level measurement system. A laser diode (TLS001-635, Thorlabs, Newton, NJ, USA), with a wavelength of 635 nm and a launched power of 1 mW, was used to generate a laser beam. A photodiode (S120, Thorlabs, Newton, NJ, USA), with a responsivity of 0.41 A/W at 635 nm and a resolution of 1 nW, was used to detect light signals at the POF output end. The POF probe was fixed straight on a ruler and placed vertically inside the beaker. The V-groove structure faced the opposite side of the ruler. Glycerin solutions with different concentrations were used as specimens to examine the sensor performance. Refractive indices were measured by an Abbe refractometer and described by the relation of n = 1.33 + 0.13*C*, where *C* is the volume concentration of the glycerin. The experiment was performed at a room temperature of 25 °C.

**Figure 4:**
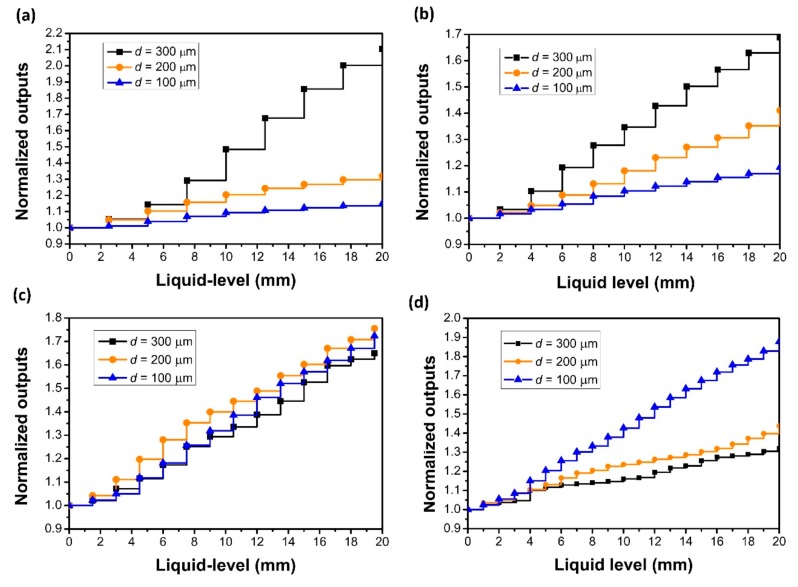
Sensing performances for POF probes with different V-groove pitches and depths, where the V-groove pitches are 2.5 (**a**), 2.0 (**b**), 1.5 (**c**), and 1.0 mm (**d**), and the V-groove depths are about 100, 200, and 300 μm, respectively. The V-groove angle is 60°, and the refractive index of the liquid is 1.39.

The influences of the V-groove depth and pitch of the POF probes on the liquid level sensing performances were investigated, as shown in Figure 4a–d, which presents the relation between the normalized outputs and liquid level for the probes with different V-groove pitches of 2.5, 2.0, 1.5, and 1.0 mm, and with the V-groove depths of 100, 200, and 300 μm, respectively. The V-groove angle of the probes was 60°. The uncertainties of the V-groove pitch, depth, and angle were ±20 μm, ±10 μm, and ±3°, respectively. The glycerin solution with a refractive index of 1.39 was used as the specimen. The normalized output power equals P_i_/P_0_, where P_i_ and P_0_ are the output power measuring in the glycerin solution and in air, respectively. The sensitivity is determined as the ratio of the normalized output power variations to the corresponding liquid level changes. The liquid level position before the first V-groove structure was set as the zero-level position. From the experimental results, it was found that, when the V-groove structure was immerged by the liquid, the output power increased, while as the liquid level changed at the straight POF sections, it remained stable; thus, the response curves of the sensor probes appear as a step ladder with rising liquid levels.

The sensitivity results from Figure 4 are shown in Table 1. It is found that the highest sensitivity for the probe with a V-groove pitch of 2.5 mm is obtained when the V-groove depth is 300 μm; however, for the probe with a pitch of 1 mm, the highest sensitivity is obtained when the V-groove depth is 100 μm. It indicates that the sensitivity depends on both the V-groove pitch and depth. To obtain a higher sensitivity for a larger V-groove pitch, the optimized depth should be large, otherwise, the depth should be small. This may be because as the V-groove pitch decreases or the V-groove depth increases, the transmission loss will increase, which may lead to a lower sensitivity. The sensitivities obtained here are much higher than that in the previous report of [15]. It is also found that the sensor resolution is determined by the V-groove pitch. A higher resolution could be obtained from a small V-groove pitch.

**Figure 5:**
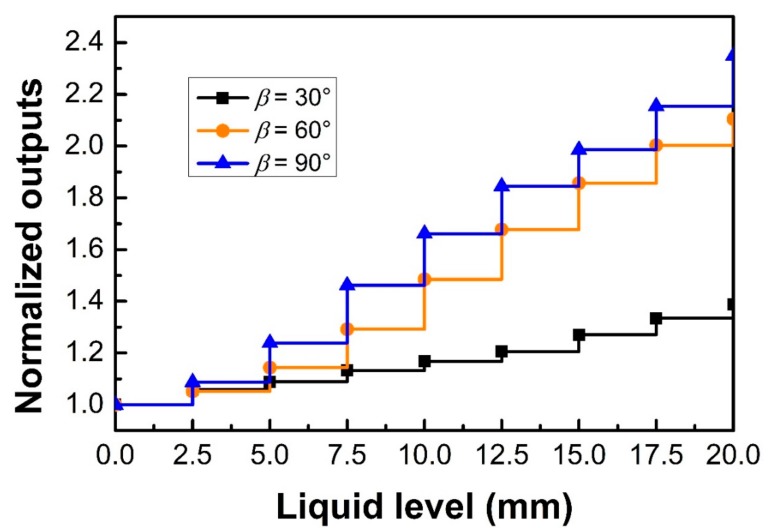
Sensing performances for POF probes with different V-groove angles of 30°, 60°, and 90°, where the V-groove pitch and depth are 2.5 mm and 300 μm, respectively, and the refractive index of the liquid is 1.39.

The influence of the V-groove angle on the liquid level sensing performances was investigated, as shown in Figure 5. The V-groove pitch and depth of the tested sensing probe were 2.5 mm and 300 μm, respectively. Additionally, the V-groove angles were 30°, 60°, and 90°, respectively. The refractive index of the liquid was 1.39. The results from Figure 5 are shown in Table 2. It is found that the probe with a V-groove angle of 90° had the highest sensitivity of 0.0698 mm^−1^. This may be because more light power will be modulated for the probe with a V-groove angle of 90°.

**Figure 6:**
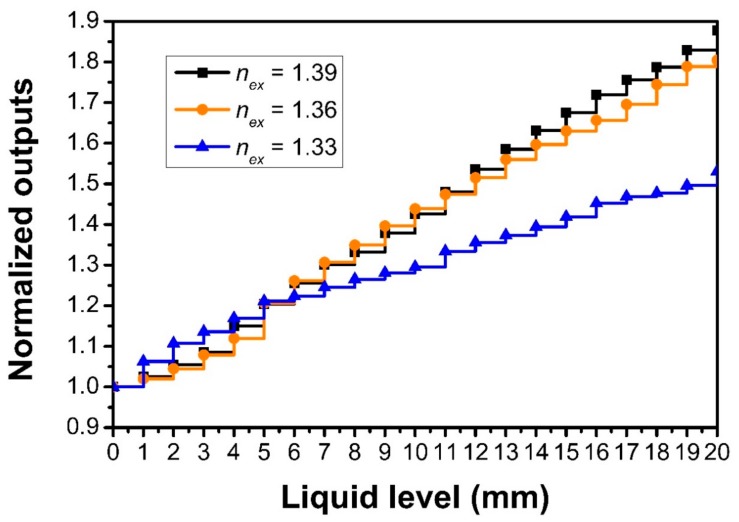
The sensor responses for testing liquids with refractive indices of 1.33, 1.36, and 1.39, respectively, where the V-groove pitch, depth, and angle of the sensor probe are 1 mm, 100 μm, and 60°, respectively.

Figure 6 shows the influence of the liquid refractive index on the sensing performance for the probe with a V-groove pitch of 1 mm, depth of 100 μm, and angle of 60°. Glycerin solutions with different refractive indices of 1.33 (water), 1.36, and 1.39 were prepared as the specimens. We note that the sensor sensitivity and response change slightly for the mediums with refractive indexes from 1.36 to 1.39. The experimental results also show that the linearity of the sensor response declines when the testing liquid is water, while for a low liquid level before 5 mm, the sensitivity is high, and after that, the sensitivity is decreased. This might be because the V-groove depth is inconsistent and the coupling efficiency is weaker for water whose refractive index is low. The results from Figure 6 are shown in Table 3.

**Figure 7:**
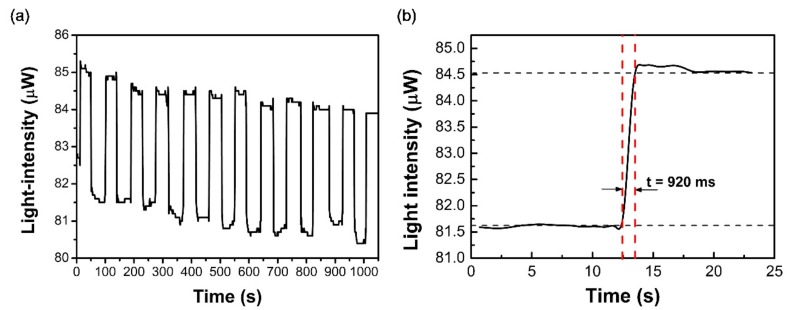
The dynamic performance (**a**) and the measured response time (**b**) of the sensor where the V-groove pitch, depth, and angle of the sensor probe are 1 mm, 100 μm, and 60°, respectively, and the refractive index of the testing liquid is 1.39.

We made the testing liquid level rise and fall repeatedly to examine the sensor dynamic performance. The V-groove pitch, depth, and angle of the sensor probe were 1 mm, 100 μm, and 60°, respectively. The refractive index of the testing liquid was 1.39. The output powers were logged continuously when the liquid level rose or fell, as shown in Figure 7a. The experimental results showed that the repeatability of the sensor was satisfactory. The response time was calculated as the time interval from the beginning of the change to the new point of stability, as shown in Figure 7b. Experimental results demonstrate that the sensor has a quick response, and the response time is about 920 ms. Thus, it is much faster than those in the reports of [3,14], which are about 180 s and 120 s, respectively. The limitations of the response time for the sensor may be associated with the response of the change in the liquid level. The burrs of the response curve may be caused by both the fluctuation of the light source and the flowing of the hanging liquid at the V-groove regions. It is also found that during subsequent modulation of the liquid level, the signal decreases. This may be caused by fluctuation of the light source and noise of the detector. Therefore, a more stable light source and a lower noise detector are recommended for this sensor.

**Figure 8:**
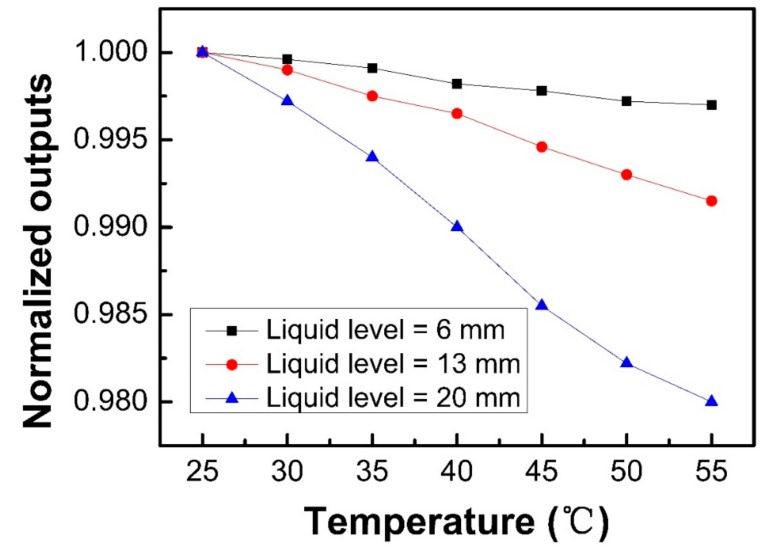
The temperature dependence of the sensor with different liquid levels of 6, 13, and 20 mm, respectively, where the refractive index of the liquid was 1.39 and the V-groove pitch, depth, and angle of the sensor probe were 1 mm, 100 μm, and 60°, respectively.

The temperature effect of the sensor was also tested, as shown in Figure 8. The test was carried out with different liquid levels of 6, 13, and 20 mm, respectively. The refractive index of the liquid was 1.39 and the temperature was changed from 25 °C to 55 °C, with a step of 5 ± 1 °C. The V-groove pitch, depth, and angle of the sensor probe were 1 mm, 100 μm, and 60°, respectively. The normalized output power equals P_t_/P_25_, where P_t_ and P_25_ are the output power measuring the temperature of t °C and 25 °C, respectively. The experiment results showed that as the temperature increases, the sensor output decreases. It is also found that the more the V-groove structure was immerged, the more obvious the temperature effect was, and when all the V-groove structure was immerged by the liquid, the sensitivity to temperature was 0.0007 °C^−1^, which was about two orders lower than the one to the liquid level. This is because the influence of temperature on the sensing performance can be divided into two aspects [17]. One is the thermal optic effect of the testing liquid, which is the higher the liquid level measures, the more significant the effect is. The other aspect is the thermal optic effect and thermal expansion effect of the sensor probe, with the material properties and the numerical aperture of the POF able to be altered by changes of temperature. The two aspects will cause a subtle change in the output power, resulting in a measurement error. However, it is easy to distinguish output changes caused by liquid level and temperature variations of the proposed sensor, for when the V-groove region is immerged or emerged by the liquid, a quick and sharp output change will be found, while for the temperature variation, the response time of the sensor is long. Therefore, the influence of temperature in the moderate range could be ignored for this liquid level sensor. However, if the change of temperature is too high, for example over 70 °C, the temperature effect must be considered, for the transmission properties of the light in the POF will be severely affected.

## 4. Conclusions

We have presented a high sensitivity and easily fabricated liquid level sensor based on a POF with a V-groove structure. The sensing probe was fabricated by the die-press-print method, which effectively reduces the complexity of the fabrication process and makes it easier for mass production of liquid level sensors. This process increases the liquid level sensitivity of the sensor. The influence of the structural parameters, such as the V-groove pitch, depth, and angle, on the sensing performance were investigated experimentally. Experimental results showed that the sensitivity could be improved by changing the structural parameters. When the V-groove pitch was 2.5 mm, the V-groove depth was 300 μm, and the V-groove angle was 90°, the sensitivity reached 0.0698 mm^−1^ for liquid with a refractive index of 1.39. The resolution of the proposed sensor was determined by the V-groove pitch. The sensor performance for liquids with different refractive indexes and the sensor dynamic response were also tested. The results demonstrated that the sensor sensitivity and response were similar for mediums with a refractive index ranging from 1.36 to 1.39, and a fast response time of 920 ms was obtained. Finally, the temperature dependence was analyzed, when all the V-groove structure was immerged by the liquid, the sensitivity to temperature was 0.0007 °C^−1^, which was about two orders lower than the one to the liquid level. Therefore, it is easy to distinguish the responses of temperature and liquid level changes. The sensor is a low-cost solution for detecting the liquid level, and its fabrication method can be used for other sensing applications.

## Figures and Tables

**Table 1:** Sensitivity results (unit: mm^−1^) from Figure 4.

	Depth (μm)	100	200	300
Pitch (mm)				
**2.5**		0.0078	0.0163	0.0606
**2.0**		0.0098	0.0210	0.0363
**1.5**		0.0398	0.0393	0.0359
**1.0**		0.0457	0.0195	0.0155

**Table 2:** The results coming from Figure 5.

V-groove angle (°)	30	60	90
**Sensitivity (mm^−1^)**	0.0188	0.0606	0.0698
**Correlation Coefficient R^2^**	0.9884	0.9842	0.9944

**Table 3:** The results (unit: mm^−1^) from Figure 6.

**Refractive index**	1.33	1.36	1.39
**Sensitivity (mm^−1^)**	0.0245	0.0425	0.0457
**Correlation Coefficient R^2^**	0.9801	0.9934	0.9956
